# Understanding the value of rehabilitation: Perspectives from South African Stakeholders

**DOI:** 10.4102/ajod.v13i0.1406

**Published:** 2024-07-19

**Authors:** Rentia A. Maart, Dawn V. Ernstzen, Gubela Mji, Linzette D. Morris

**Affiliations:** 1 Department of Rehabilitation and Health Sciences, Stellenbosch University, Cape Town, South Africa; 2 Division of Physiotherapy, Department of Interdisciplinary Health Sciences, Faculty of Medicine and Health Sciences, Stellenbosch University, Cape Town, South Africa; 3 Division of Disability and Rehabilitation Studies, Department of Global Health, Stellenbosch University, Cape Town, South Africa; 4 Department of Rehabilitation Sciences, College of Health Sciences, Qatar University, Doha, Qatar

**Keywords:** value-based care, value, rehabilitation, South Africa, rehabilitation stakeholders, disability

## Abstract

**Background:**

The need for rehabilitation in South Africa has doubled between 1990 and 2017 and is expected to increase in the coming years. However, the rehabilitation needs of South Africans (and globally) remain largely unmet. Establishing a common understanding of the value of rehabilitation can inform clinical practice and policymaking to achieve Universal Health Coverage (UHC).

**Objectives:**

This study aims to explore the value of rehabilitation services in South Africa’s public healthcare sector by gathering perspectives from stakeholders. The goal is to inform policy decisions related to the implementation of National Health Insurance (NHI) in South Africa.

**Method:**

The study used a phenomenological approach and interpretivist paradigm. Semi-structured interviews were conducted face-to-face, online, or telephonically with 12 stakeholders from various rehabilitation sectors. The value of rehabilitation was analysed and categorised into five main categories: context, service delivery, patient outcomes, economic and financial components, and collaboration within and between sectors.

**Results:**

The value of rehabilitation was found to be multifaceted, because of the varying health, economic, and social challenges faced by many South Africans.

**Conclusion:**

The study identified components of value-based rehabilitation that should be prioritised in the proposed NHI of South Africa. Future research should explore all stakeholder perspectives, including patients, and provide empirical evidence of rehabilitation’s economic and societal value.

**Contribution:**

We highlight priority areas that are central to the value of rehabilitation in South Africa and other low- and middle-income countries (LMICs). Tailoring rehabilitation services to patient and community needs is crucial for achieving value-based care. Given South Africa’s commitment to the United Nations Convention on the Rights of Persons with Disabilities, prioritising rehabilitation remains essential.

## Introduction

The healthcare industry globally is increasingly emphasising the concept of ‘value’. Various stakeholders, including healthcare funders, service providers, academics, and organisations, recognise the necessity for more efficient healthcare delivery with limited resources amid rising demand (Jette 2018; WHO 2021). This recognition is part of a broader trend in healthcare, shifting from volume-based to value-based services. Volume-based services involve paying providers based on the quantity of services delivered (fee-for-service payments) (National Department of Health 2017), while value-based services prioritise quality, outcomes, efficiency, and cost (Kamal, Lindsay & Eppler 2018; Modica 2020).

South Africa is restructuring its health system with the National Health Insurance (NHI). The NHI aims to provide quality and equitable healthcare for all (National Department of Health 2017), aligning with the World Health Organization’s (WHO) value-based health services framework (WHO 2021). The NHI focusses on improving health coverage and providing equitable, quality healthcare while managing costs (National Department of Health 2017). It is therefore expected to enhance healthcare and provide value-based healthcare.

The demand for rehabilitation in South Africa is growing because of the quadruple burden of disease – human immunodeficiency virus (HIV) and tuberculosis, chronic illnesses and mental health, injury and violence, and maternal, neonatal, and child mortality (Achoki et al. 2022). Although literature reports that globally, the need for rehabilitation has increased by 63% since 1990, potentially impacting 2.41 billion people (Cieza et al. 2020), the data on the rehabilitation needs of South Africans remain scant and under-reported (Morris et al. 2019). However, it is estimated that the associated years lived with disability (YLD) in South Africa has almost doubled from 1990 to 2017, and is expected to increase by 17% from 2017 to 2022 (Louw et al. 2021). Despite the estimated increase of the need for rehabilitation, it is often treated as an add-on service, lacking sufficient policy support from the government and leading to undervaluation and under-prioritisation (WHO 2017a).

For people with disabilities (PWDs), rehabilitation forms an essential part of their well-being and their participation in life (Health Systems Trust 2020; Mlenzana et al. 2013; WHO & The World Bank 2011). However, because of the lack of access to healthcare, many PWDs forfeit the opportunity to receive rehabilitation (Kahonde, Mlenzana & Rhoda 2017; WHO 2015). South Africa has ratified the United Nations Convention on the Rights of People with Disabilities (UNCRPD) and supports the Sustainable Development Goals (SDGs) that aim to guide global development (Statistics South Africa 2019). These commitments are focussed on ensuring equitable access to services and inclusion in decision-making for PWDs. Furthermore, South Africa’s commitment to Universal Health Coverage (UHC) aims to provide quality healthcare, including rehabilitation, to all citizens without financial hardship (WHO 2017a), and therefore the government needs to prioritise rehabilitation services in South Africa.

Rehabilitation, defined as a ‘a set of interventions designed to optimize functioning and reduce disability in individuals with health conditions in interaction with their environment’ (World Health Organization 2023), is generally accepted as a person-centred service aiming to improve or restore individual function and reintegration into society (WHO 2017b, 2018; Wade 2021). Despite growing interest in value-based healthcare (VBHC), limited literature explores the value of rehabilitation (Jordan & Deutsch 2021; Louw, Dizon et al. 2020; Morris et al. 2019; Roth & Hornby 2022). Jewell, Moore and Goldstein (2013) advocated for the value of physical therapy, emphasising best practices, performance measurement, and cost-effectiveness evaluations. Rundell et al. (2015) proposed a framework for stakeholders to define rehabilitation’s value, considering outcomes such as quality of care, clinical outcomes, patient and caregiver satisfaction, and costs. In a recent study, Jordan and Deutsch (2021) propose measuring rehabilitation value through cost-effectiveness analyses across provider, health system, and societal perspectives, associating value with costs and effectiveness. They suggest a budget impact analysis for shorter-term cost assessments (1–3 years) (Jordan & Deutsch 2021). Other frameworks for value, specific to occupational therapy in Singapore (Wong et al. 2022) and physiotherapy (Cook et al. 2021) highlight aspects such as personalised goal-setting, meaningful outcomes, managing costs, patient-centred care, guideline-concordant integrated care, cost-effectiveness, patient experience, and outcomes. The literature reveals varying interpretations of value, reflecting different perspectives and circumstances among stakeholders.

South Africa’s restructuring of the health system presents an opportunity to examine the value of rehabilitation. As the world moves towards VBHC and UHC, it’s important to define and determine the value of rehabilitation services to be able to inform policy decisions and strategic health package purchasing. A recent systematic review of VBHC from healthcare professionals’ (HCPs) perspective revealed many components that need to be considered in value-based care such as HCP, job, and environmental factors (Van Engen et al. 2022). However, none of the 45 articles used in the review were from low- and middle-income countries (LMICs). Investigating the opinions of rehabilitation stakeholders in South Africa will help LMICs understand how rehabilitation value is perceived, and which factors should be considered when providing value-based rehabilitation services. This study aims to explore the perspectives of rehabilitation stakeholders on the value of rehabilitation in the public health sector of South Africa.

## Research methods and design

### Research design

A descriptive qualitative study with a phenomenological approach using an interpretivist paradigm was conducted (eds. Ritchie & Lewis 2003). The authors explored the phenomenon of value-based rehabilitation services as understood by different stakeholders in various settings such as clinical, academic, government or managerial settings, based on their experiences.

### Setting

This study was conducted in South Africa, a culturally and geographically diverse country with nine provinces. Each province has its own legislature, premier and executive council (Department of Government Communication and Information System 2023). Healthcare in South Africa is governed by the National Department of Health (NDoH). The role of the NDoH is policy formulation, monitoring and evaluation, and provision of support and coordination to the Provincial DOHs (The Presidency: Republic of South Africa 2004). Each Provincial DOH is mandated to organise and deliver healthcare services to the province (Katuu 2018).

In South Africa, healthcare, including rehabilitation, is provided at all levels of care, which entails primary healthcare (PHC) services as well as healthcare provided at district, regional, tertiary, and central hospitals, each with a specific purpose (NDoH 2000, South African National Department of Health 2015; Western Cape Government: Health 2014). The South African healthcare system consists of two sectors, namely the public healthcare sector (serving more than 80% of the population) as well as the private healthcare sector, which provides healthcare to the minority of the population who can afford to belong to medical schemes or pay out-of-pocket healthcare costs (Burger & Christian 2020). This study focussed on the public healthcare sector because of the vast inequities that persist within the South African healthcare system with most of the population being serviced by the public healthcare sector. In addition, the NHI will ultimately lead to a restructured health system in which a single publicly owned and administered purchaser will purchase healthcare services on behalf of everybody (NDoH 2017).

### Population and sampling

#### Population: Inclusion and exclusion criteria

Key informants with a national, provincial, and/or local standing position specifically in relation to rehabilitation and disability in South Africa, were invited to share their perspectives. Rehabilitation stakeholders who only practised or were solely involved in private healthcare settings were excluded from this study. This study purposively did not include patients or end users of rehabilitation services, as the principal investigator (PI-RM), intended to explore patients’ perspectives in a separate future study.

#### Sampling

A two-step sampling process was applied as detailed next.

**Step 1: Purposive sampling:** We selected a purposive sample of stakeholders based on their knowledge, interest, and willingness to share their experiences. The selection process involved discussions with rehabilitation and disability researchers and the Ph.D. candidate’s supervisory team. Participants included a disability sector representative, a clinician engaged in rehabilitation research, national policy stakeholders, and an academic with a special interest in disability and rehabilitation services, who had published peer-reviewed articles.

**Step 2: Snowball sampling:** The primary sample, selected through purposive sampling, nominated professionals from different sectors including academia, policymaking, clinical practice and rehabilitation management, via snowballing. This approach helps when the population is dispersed and the required characteristics for the study are not easily available (eds. Ritchie & Lewis 2003). The authors anticipated that a diverse range of rehabilitation stakeholders with insights into the South African rehabilitation context would be included in this study.

#### Sample size

In the case of phenomenological research, it was estimated that 10 interviews would suffice if the interviews have produced enough in-depth data to ensure that the objectives of the study were reached (Moser & Korstjens 2018). The recruitment process in this study aimed to recruit enough participants that would lead to data saturation (a point where ‘no new information added is expected to enhance or change the findings of the study’ [Moser & Korstjens 2018]).

**FIGURE 1:**
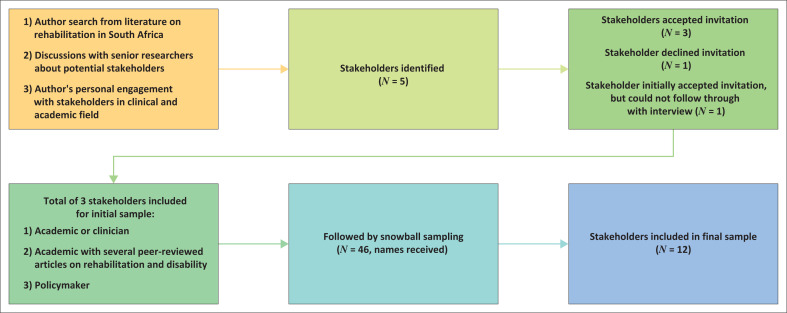
Organogram illustrating the sampling process.

Following the purposive sampling and snowballing processes, 46 rehabilitation stakeholders were invited to participate in the study. However, only 12 rehabilitation stakeholders agreed to participate in the study. Of all the nominees, only one nominee was not a healthcare professional (HCP), but a representative from the disability sector. Unfortunately, this nominee could not participate in the study because of unavailability to conduct an interview. This resulted in only HCPs participating in the study. The reasons for non-participation included a lack of response after follow-up emails were sent to the participants who initially indicated their willingness to participate (*n* = 9), non-response from 19 stakeholders, conflict of interest (*n* = 1), as well stakeholders (*n* = 3) declining to participate because of retirement, involvement in coronavirus disease 2019 (COVID-19)-related activities and time constraints, respectively. Figure 1 illustrates the sampling process in the study.

#### Demographic information of participants

A short demographic questionnaire was developed by the authors and included information about the stakeholders’ current role and workplace, years of experience, areas of expertise and/or experience, the stakeholders’ involvement in any physical rehabilitation at the time of the interview, and in what way. The purpose of this questionnaire was to contextualise the findings of the interviews and to monitor and ensure diversity in sampling.

Table 1 provides demographic information of the participants, including the area of expertise within the field of rehabilitation, their profession, years of experience, and the province where they were located during the interview period. Many of the participants had more than one area of expertise. Most of the participants were physiotherapists (PTs) (*n* = 7), followed by occupational therapists (OTs) (*n* = 4), and then speech-language and hearing therapists (SLTs) (*n* = 1).

**TABLE 1:** Demographic information from rehabilitation stakeholders.

Participant	Background	Area of expertise	Years of experience in rehabilitation	Province
P1	PT	Clinical and academic	23	WC
P2	OT	Policy	32	Gauteng
P3	PT	Academic	30	WC
P4	PT	Clinical and policy and management	18	KZN
P5	ST	Management and Clinical	28	Mpumalanga
P6	OT	Clinical	29	Gauteng
P7	OT	Academic and policy	50	KZN
P8	OT	Management and policy	21	EC
P9	PT	Academic	24	Gauteng
P10	PT	Academic	32	WC
P11	PT	Academic	25	WC
P12	PT	Academic and clinical	5	WC

PT, Physiotherapist; OT, Occupational Therapist; ST, Speech Therapist; WC, Western Cape; KZN, KwaZulu-Natal; EC, Eastern Cape.

### Instrumentation

The PI conducted semi-structured individual interviews. The format was face-to-face, by telephone or online via Zoom (Zoom Video Communications Inc, Version: 5.12.2 [9281]) or Microsoft Teams (Version 1.6.00.376 (64-bit), according to the preference of the participant. Only one interview was conducted face-to-face at the university. The rest of the interviews were conducted online or by telephone. No other parties were present during the interview process except for the interviewer (the PI -RM) and the participant.

An interview guide (Box 1) was developed using international and national literature (Malakoane et al. 2020; Morris et al. 2019; Rundell et al. 2015; South African Lancet National Commission 2019; The Lancet 2009; WHO & The World Bank 2011). The interview guide was drafted by the PI and was shared and discussed with one of the authors of this article (LM), and externally audited by a senior researcher in the field. The interview guide explored aspects of the rehabilitation landscape in South Africa (reported elsewhere) as well as how stakeholders perceive ‘value’. Box 1 contains only the questions relevant to this article about the value of rehabilitation. The interview guide was piloted on a participant before the start of the study by the PI, and the data were included in the findings of this study.

BOX 1 Interview guide questions.

How would you define the concept of ‘value’ in general?

What factors do you think are important to consider when exploring the concept of ‘value’ in terms of rehabilitation?

### Procedure

The PI contacted the initial sample and subsequent nominated peers via email. Contact details were obtained from colleagues who nominated the participants or university websites. A second email was sent to interested participants with an information sheet, consent form, and preferred interview details. Two weeks were given for a response. If the contacted participants did not respond within 2 weeks of receiving the two follow-up emails, the lack of response was accepted as an indication that the participant was not interested or available to participate in the study. In these instances, the recruitment attempt ended. The recruitment process was continuous as participating stakeholders nominated other potential participants throughout the study, which was conducted between January and November 2020. All interviews, lasting between 45 and 75 min, were conducted in English and recorded using a digital recorder or Zoom or Microsoft Teams with additional notetaking. Recordings were de-identified and transcribed. Following the analysis of 12 interviews, the PI (RM) and another author (DE) convened to determine richness of data and agreed that data saturation had been reached. Data collection concluded at this point and no additional participants were recruited.

The PI is a female physiotherapist with clinical experience in the private and public healthcare sectors, including hospitals, outpatient facilities, rehabilitation, step-down facilities, and home visits. Except for one participant, whom the PI knew in a professional capacity, the PI had no personal and/or professional relationship with any participant. The PI, a postgraduate student in physiotherapy, introduced herself to all participants and established rapport by explaining the interview process and creating a comfortable environment. The co-authors, well-known researchers in the rehabilitation community, did not participate in the interviews to protect anonymity of the participants. They were involved in coding, data analysis, and interpretation, forming the foundation of this research. The authors of this article are all female, native South Africans, and collaborated on the critical revision and drafting of this article.

### Data analysis

We used an inductive approach to generate innovative ideas. Through content analysis, we developed themes and categories that described value-based rehabilitation experiences of stakeholders in different settings. The analysis was framed within existing theories and frameworks of value-based care pertaining to rehabilitation as described earlier in the introduction (Cook et al. 2021; Jewell et al. 2013; Jordan & Deutsch 2021; Rundell et al. 2015; Wong et al. 2022). This approach helped us gain a better understanding of the phenomena under investigation (Hsieh & Shannon 2005).

#### Coding

Each author coded two interviews. After comparing our codes and categories, we created a joint codebook. The PI (RM) continued coding the rest of the interview transcripts, using ATLAS.ti, version 9. Open coding was used for the identification of new emerging codes and categories, which were then added to the codebook and discussed with two of the other authors (DE, LM) throughout the analysis process (Gale et al. 2013; Moser & Korstjens 2018). A continuous and iterative consultative process was followed throughout the coding process to ensure consensus of the coding among the authors. The PI coded all transcripts. This process of iterative data analysis and peer debriefing (Carter et al. 2014), assisted in a broader understanding of the content of the interviews and ensured a more accurate representation of the final theoretical framework (Chapman, Hadfield & Chapman 2015; Ritchie & Lewis 2003). The authors identified patterns and correlations among codes by labelling words, phrases, and sentences. The codes were grouped by frequency of occurrence and categorised under a particular theme. The team followed an iterative process with continuous discussion and consensus to finalise the codes and categories.

### Ethical considerations

The study was approved by the Health Research Ethics Committee (HREC) at Stellenbosch University, South Africa, (Ref no. S19/07/123 [PhD]). Written and verbal informed consent was obtained from each stakeholder prior to the commencement of the study. Participation was completely voluntary, and no incentives were offered to participants. The study revealed neither personal information nor stipulated any personal details when the findings were reported.

## Results

### Interview results

Two main areas were explored namely the meaning of person-centred value and the value of rehabilitation services. These areas formed the themes for this study. However, emerging categories were applied to further explore the value of rehabilitation, as indicated in Table 2. Verbatim quotes are provided with each participant’s unique reference number to explore the findings.

**TABLE 2:** Main themes and categories.

Theme	Category	Sub-category
The meaning of person-centred value	-	Fulfilling individualised needs and purpose Inclusive of others Enhancing quality of life Monetary value
Value-based rehabilitation services	Context	Patient-specific Community-specific Setting where services are provided
	Service delivery components (treatment)	Quality Evidence-based practice Efficacy and/or efficiency and/or effectiveness Optimisation Outcome measures Acceptability
	Patient outcomes	Impact and benefits Change in quality of life and health status and function Reintegration and participation
	Economic and financial components	Economic evaluations Sustainability Measurability Affordability Appropriate health indicators
	Collaboration and unity within the rehabilitation sector	Unity and consensus Advocating for the rehabilitation and disability sectors Streamlining services

### Components of value

#### The meaning of person-centred value

The participants shared their understanding of value, which was person-centred. The most common ideas (themes) included value fulfilling individualised needs, being inclusive of others, enhancing and enriching quality of life, and monetary value.

Participants felt that value is a personal or individualized experience and that it serves a particular need or purpose for that individual:

‘So, I think it’s when it’s personalized or individualized? … If something is of value to me, it’s because a few reasons, but because I specifically need it an and it will be useful to me.’ (P12, PT, Academic/clinical)

‘… it would need to be relevant to me, it would need to be consistently available or available when it is needed. … but I think something that is valuable, I guess meets my particular, would meet my particular needs whatever they are.’ (P6, OT, Clinical)

However, some participants felt that value goes beyond the individual experience and that it’s inclusive of others:

‘… something that is not just going to benefit me alone it must make a difference in people’s lives.’ (P8, OT, Management/ policy)

‘I would say that’s something that is meaningful, that it has some meaning for me personally or to others that are close to me.’ (P11, PT, Academic)

Furthermore, participants felt that value is when something enhances and enriches quality of life:

‘… so, when it allows, or it enriches my life somehow by allowing me to do something that I wouldn’t be able to do so easily in other ways. And it gives me great pleasure, ja it’s all about quality of life and pleasure, or making things more easy or possible, enabling me to do something.’ (P5, ST, Management/ clinical)

In addition, the aspect of value being associated with its monetary value was also highlighted by the participants:

‘… When it, when it serves a particular purpose that is one, and that purpose can be either in financial, in that it is expensive and when I sell it I can get money …’ (P3, PT, Academic)

#### Value-based rehabilitation services

The participants highlighted various factors that can influence the value of rehabilitation services including aspects around context in terms of the patient, the community, setting of service provision, service delivery components; outcomes of rehabilitation services, economic and financial components as well as the impact of collaboration on providing value-based rehabilitation.

**Context:** Most of the participants emphasised the importance of the context of the patient when providing value-based rehabilitation services. Understanding not only the intricate needs of the patient but also how the contexts of communities may differ and how the value of rehabilitation may differ for different individuals and communities was mentioned as important considerations to enhance the value of rehabilitation services:

‘… to me value … you can’t understand value-based therapy unless you live there, unless you can be a part of that community. Then you can understand what is valuable for that community … maybe it is very different to what is valuable in another community somewhere else.’ (P7, OT, Academic/policy)

‘And then the other one is the value to the patient that we need to look at. How did they value it in terms of improving their participation in society and their quality of life?’ (P3, PT, Academic)

‘The voice of the person, client centeredness, you know what is it that that that that the patients or the person or the people with disabilities or impairments, Or what is it that they find valuable?.’ (P10, PT, Academic)

**Service delivery components:** The participants expressed that for rehabilitation services to be of value, it needs to be evidence-based, acceptable to the people, and it needs to be of high quality. Effective, efficient, and efficacious treatment that optimises the patient’s treatment sessions were other aspects that must be considered in value-based rehabilitation as described by participants. Additionally, participants emphasised the importance of using appropriate outcome measures to demonstrate the value of rehabilitation services and being held accountable for delivering quality rehabilitation services:

‘Effectiveness of the treatment, and efficiency of the treatment. So, I can move from one level to the next in a short space of time if I have it targeted and focused, right?.’ (P1, PT, Clinical/ academic)

‘Obviously outcome measures. Trying to get therapists to do outcome measures …’ (P4, PT, Clinical/policy/management)

‘Well, I think to me what is important, is focusing on outcomes and looking at the efficacy of whatever interventions, looking at the evidence associated with whatever intervention.’ (P2, OT, Policy)

‘And it must be acceptable to people, and it must have that certain passion to the patients that you are providing the service to and the quality, it must be of good quality.’ (P8, OT, Management/policy)

‘I think in a public health context, particularly in a primary health care level, I always preach to my students: do a screening assessment and treat during your first session. Do not send this person away without value from that one session.’ (P5, ST, Management/clinical)

**Patient outcomes:** The participants also mentioned different outcomes that may influence the value of rehabilitation. Participants believe that creating a difference in quality of life and/or function and health status as well as facilitating reintegration into home, work, and social life are important considerations in providing value-based rehabilitation:

‘So, we are looking at maybe changes in health-related quality of life, changes in functioning … and I don’t think we are doing enough of that [research] … no evidence of what we are doing and that is a big gap in rehabilitation. We can’t say what our value is.’ (P3, PT, Academic)

‘I think about re-integration back into the home, back at work. So, have you made a difference to that person’s participation, that person’s functional abilities?.’ (P1, PT, Clinical/academic)

In addition, it was also mentioned that value-based rehabilitation services can restore purpose and dignity in the lives of people who require rehabilitation:

‘Yeah, so it’s just that sense of purpose, independence, and quality of life.’ (P9, PT, Academic)

‘… they place value … in being able to care for their families, for example financially and so we need to be able to, to get them to a point where at least there’s some value in dignity in being able to earn some sort of …’ (P10, PT, Academic)

**Health economics and financial components:** Various factors related to an economic or financial bearing that may impact the delivery of value-based rehabilitation services, were mentioned by the participants. These factors include performing economic evaluations, considering financial and time costs, input from the providers and caregivers, and ensuring the measurability and sustainability of rehabilitation services:

‘You can do a what they would call a cost-benefit analysis versus a cost-effective analysis.’ (P3, PT, Academic)

‘And I think of cost should be perceived cost as well, sort of cost-benefit and I think it comes also in terms of if you look at not just financial cost but time cost and commitment by caregivers.’ (P5, ST, Management/clinical)

‘… that has to be sustainable….’ (P12, PT, Academic/clinical)

**Collaboration and unity within the rehabilitation sector:** The participants mentioned various aspects in terms of collaboration to strengthen the value of rehabilitation services. They alluded to the impact of strong collaborative efforts between various stakeholders to streamline services and the importance of the disability and rehabilitation sectors advocating for each other in value-based rehabilitation care:

‘… but I feel that there is an overlap and that we can both [*the rehabilitation and disability sectors*] be advocating for one another on this [*the value of rehabilitation*].’ (P4, PT, Clinical/policy/management)

‘I think that if there was a way to streamline services or manage our services slightly differently … but collaborate with other players, other stakeholders, other members of the health team to add value to that one monthly visit, I think that that would be really, really important.’ (P6, OT, Clinical)

A common understanding of what rehabilitation entails was important for participants in providing value-based rehabilitation services:

‘… if we talk about value is, first of all is that we need to know what is rehabilitation. Like what, what it is. We need uniformity and understanding of what it is, because you can’t actually put value to something that is vague and elusive.’ (P11, PT, Academic)

Besides working with rehabilitation stakeholders, a participant also mentioned the importance of public participation and how the value of rehabilitation can be further enhanced if understood and appreciated by the public:

‘It needs to be understood and appreciated by the general public that value, you understand?’ (P11, PT, Academic)

## Discussion

This study investigates the perspectives of rehabilitation stakeholders concerning the value of rehabilitation services within South Africa’s public healthcare sector. The findings reveal nuanced interpretations of ‘value’ at the individual level, influenced by contextual variables. Participants associate ‘value’ with enhancing quality of life, meeting needs, achieving goals, and providing peace, happiness, and pleasure, occasionally incorporating monetary considerations. The value of rehabilitation services is intricately tied to various factors, including the patient’s setting, community dynamics, and service provision. Emphasising high-quality, evidence-based rehabilitation, particularly in primary healthcare settings, is imperative. Key outcomes, such as improved quality of life and the restoration of patients’ roles in the home, work, and social spheres, play a pivotal role. Participants agree that value-based rehabilitation is manifested through sustainable, measurable practices, evaluated through economic assessments and robust health indicators. Effective collaboration among diverse rehabilitation stakeholders, including the public, is deemed crucial.

Participants’ feedback indicates a link between personal values and the perception of rehabilitation value, as both are associated with improving various life aspects. This suggests diverse implications of value for individuals in different circumstances (Wong et al. 2022). Despite this diversity, a shared understanding among rehabilitation stakeholders about the essence of the value of rehabilitation can facilitate knowledge translation into practice, enabling the delivery of value-based care to those in need.

Value-based healthcare is defined differently in the literature (Steinmann et al. 2020). Porter defines value as patient outcomes relative to total costs (Porter 2010). Porter’s value equation is arguably the most commonly used proposition of VBHC (Talluri, Harrington & Halawi 2020); however, Hurst et al. (2019) are of the opinion that the value equation defined by Porter does not consider the allocation and distribution of resources in a health system funded through taxation or social insurance such as the National Health System. Teisberg, Wallace and O’Hara (2020) say value can only be assigned to improving patient health outcomes and not just quality (Teisberg et al. 2020). Steinmann et al. (2020) explain that the context influences how VBHC is understood, making it ambiguous. Similarly, the WHO emphasises the importance of countries identifying the needs of the community to be able to deliver value-based care (WHO 2021). This is in agreement with our findings as participants highlighted that context is particularly important in South Africa. Despite being classified as an upper-middle-income country, South Africa ranked as the most unequal nation globally in 2022 (The World Bank 2022). This inequality is primarily attributed to inherited circumstances, beyond an individual’s control, encompassing factors such as location, gender, age, parental background, and race (The World Bank 2022). This inequality significantly impacts access to healthcare, including rehabilitation.

The challenges to rehabilitation access in South Africa include limited capacity at the PHC level, a scarcity of specialised rehabilitation facilities, and early hospital discharges to accommodate critically ill patients (Louw et al. 2023). Additionally, access problems for those in rural areas stem from socio-economic and geographic factors, including rural terrain, poor road conditions, long distances to healthcare facilities, transportation deficits, high out-of-pocket expenses, and prevalent poverty and unemployment (Vergunst et al. 2017; Health Systems Trust 2020). This is particularly problematic for PWDs as a lack of access to rehabilitation can result in worse health outcomes, deterioration in function and could add to the disability-poverty nexus when reintegration and participation in education and work is hampered (Health Systems Trust 2020; Sherry 2015; South African Human Rights Commission 2017). While these challenges are not unique to South Africa, the commitment to UHC in the country makes our findings relevant for the implementation of value-based care, ensuring inclusivity in healthcare access. As a crucial component of addressing PWDs health needs, rehabilitation should be prioritised within health systems and funding decisions.

The South African Department of Health (DoH) is dedicated to achieving UHC through the NHI implementation. Value-based health care is deemed a core component for UHC, ensuring financial protection, quality healthcare, and equitable access aligning with UHC goals (WHO 2021). Participants suggested that economic evaluations are conducted to show the benefits of rehabilitation and emphasised the imperative of quantifying these benefits, to support the value of rehabilitation. Economic evaluations have been recommended in the literature to illustrate the value of rehabilitation (Jewell et al. 2013; Jordan & Deutsch 2021; Rundell et al. 2015). Scholars in South Africa have already started with such evaluations. Louw et al. (2020) reported on the cost-saving benefits of stroke rehabilitation for previously employed people who suffered a stroke and focussed on return to work as the outcome. The study illustrated that a work intervention programme could result in a net saving (in terms of tax recovery and savings from disability payments) of R133.1 million over 5 years, compared to the costs of the programme, survivor rates and return-to-work rates, which as the researchers suggested, should be considered as an investment for the country (Louw et al. 2020). The WHO’s value-based health services framework emphasises a value-for-money component alongside integrated people-centred health services (WHO 2021). While the economic component aids in understanding rehabilitation service value’s impact on the economy, a patient-centred approach, considering outcomes important to the patient, remains essential for a comprehensive evaluation. Visagie and Swartz (2018) reports that the needs of marginalised people such as PWDs are often overlooked even in a country like South Africa where human rights and an inclusive approach are highly prioritised. Furthermore, Roth and Hornby (2022) state that when considering the definition or measurement of the value of rehabilitation, it is important to include the inputs and perspectives of people living with a disability. It therefore remains crucial to conduct research on the value of rehabilitation from the patient and/or client’s, as well as the community’s perspective to be able to understand what matters most to the patient and the community, and to foster inclusivity.

Our study underscores the significance of patient outcomes in evaluating rehabilitation. These outcomes, that go beyond economic considerations, encompass quality of life, function, reintegration, dignity restoration, and societal benefits, aligning with existing literature highlighting the positive impact of rehabilitation on end-users including PWDs (WHO 2017c; McClure & Leah 2021). However, rehabilitation does not only address health issues but it also impacts the economy at large through cost savings (e.g., decreased hospital admissions or readmissions) as well as facilitating independent healthy lives of productive members of the community (WHO 2017c). Notably, our findings emphasise the responsibility to illustrate the impact and benefits of rehabilitation within the South African context. In a LMIC, such as South Africa where resources are limited, but the need for rehabilitation continues to rise, it is important to ensure that rehabilitation services are cost-effective without compromising on achieving the best possible outcomes for the end-user of rehabilitation. Literature suggests that providing evidence on health economic measures is crucial for demonstrating the economic and societal advantages of rehabilitation to government, policymakers, and funders (Louw et al. 2020; Neill et al. 2023). Measuring and recording outcomes not only enhance the value of rehabilitation but also hold providers accountable for treatment results (NdoH 2017). This approach ensures that end-users receive quality care aligned with their goals and desires, resulting in benefits for all stakeholders.

Porter (2010) asserts that ‘value’ in healthcare encompasses crucial aspects such as quality, efficiency, evidence-based practice, and safety. Consistent with Porter’s perspective, our participants emphasise that service delivery factors, including efficient and effective evidence-based practice of high quality, are essential for providing value-based rehabilitation services. Evidence-based practice integrates scientific information from research studies, clinician expertise, and consideration of patient values and desires (Roth & Hornby 2022). Clinical Practice Guidelines (CPGs) serve as effective tools offering evidence-based practice recommendations to diverse stakeholders, including rehabilitation professionals (Dizon et al. 2018). Various value frameworks for physiotherapy have highlighted the importance of CPGs in delivering value-based care (Cook et al. 2021; Jewell et al. 2013). However, literature indicates a scarcity in the development, relevance, and implementation of allied health CPGs in South Africa (Dizon et al. 2018). Jewell et al. (2013) stress the need for a collaborative effort within the profession to demonstrate the value of physical therapy and prevent professional irrelevance. Hence, it is crucial for the South African rehabilitation sector to collaborate in developing and implementing relevant practice guidelines. This collaborative effort can enhance the value of rehabilitation through the provision of effective, efficient evidence-based practice. A good example of such a collaborative effort is the Acute Hospital Rehabilitation Intensive Services (ARISE) project that provide acute, multi-disciplinary, comprehensive rehabilitation for stroke patients to ensure enhanced short- and long-term outcomes based on the International Classification of Functioning, Disability and Health (ICF) framework (Langton-Frost et al. 2023).

### Summary

**FIGURE 2:**
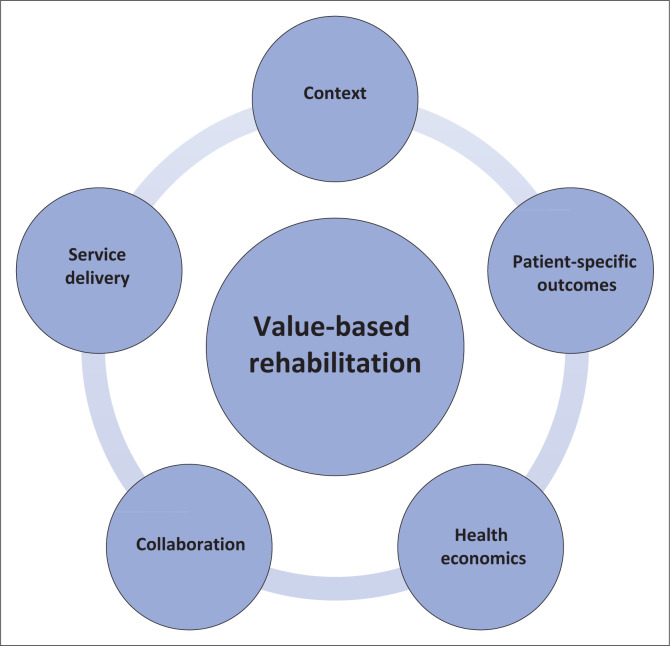
Components of value-based rehabilitation services in South Africa.

In summary, our research underscores the unique value of rehabilitation in the South African context, shaped by diverse health, socio-economic, and socio-geographic challenges faced by its citizens. While similar challenges are present in other LMICs, the impact on value-based rehabilitation may be comparable. South Africa’s commitment to UHC emphasises every citizen’s entitlement to quality healthcare. Achieving UHC is contingent on providing value-based care (WHO 2021). The participants identified five crucial components essential for determining the value of rehabilitation in South Africa, as depicted in Figure 2.

Prioritising these components within the healthcare system, clinical practice, and policy is imperative to promote and foster value-based rehabilitation services in South Africa and ensure that the rehabilitation needs of PWDs are addressed. For example:

- priority should be given to improve access to rehabilitation at the PHC level (Louw et al. 2023) (*context*)
- to ensure that rehabilitation end users can access quality rehabilitation (*service delivery*) close to where they live, work, or go to school (WHO 2018)
- to achieve the outcomes that are desirable to them (*patient outcomes*) (Porter 2010) in keeping with evidence-based practice
- which will result in a positive economic impact by limiting out-of-pocket costs (WHO 2017c) for the patients and providing individuals who can contribute to the economy (*health economic factors*).
- this will require a collaborative effort within the rehabilitation sector between different professions, as well as across sectors such as health; housing and sanitation; and transport (NdoH 2000) *(collaboration)*.

The implementation of the NHI offers a substantial opportunity to tackle health inequities in the country and enhance rehabilitation services within the healthcare system (Sherry 2015). To effectively demonstrate the value of rehabilitation services and establish relevant health indicators, future research should prioritise the collection and presentation of empirical data. It is important to acknowledge that the value components identified by our research participants may not be universally applicable. Therefore, conducting similar studies in other settings and countries, especially in LMICs, is highly recommended to ascertain what value-based care will entail for them (WHO 2021).

### Strengths and limitations

The findings of this study contribute to the existing knowledge on value in healthcare, particularly in the context of rehabilitation services. To the best of our knowledge, no other research has explored the perspectives of stakeholders in LMICs on this subject. The study results provide a comprehensive framework for understanding the value of rehabilitation, with potential applications in shaping policy direction. A shared understanding of the value of rehabilitation among healthcare professionals can foster collective efforts to deliver value-based rehabilitation services.

While interpreting the study’s findings, it is important to acknowledge encountered limitations. The study sample included only healthcare providers, predominantly from the Western Cape province, and the sampling method covered only five out of nine provinces, although representing both rural and urban areas. Future research should aim for a more diverse inclusion of rehabilitation stakeholders, including representatives from non-governmental organisations (NGOs) and patients by using a stratified sampling approach, for example. In fact, a follow-up qualitative study is planned to explore patients’ perspectives. This is especially important given the global shift towards value-based care, emphasising patient-centred approaches. Incorporating patients’ perspectives in healthcare research is essential for a more comprehensive understanding.

Challenges were encountered in participant recruitment as the study began shortly before the outbreak of the COVID-19 in South Africa, and continued during the early stages of the pandemic. Many invited participants did not respond or were unable to participate because of COVID-19-related commitments. Some prioritised personal or work commitments during a time of uncertainty about the virus, its impact, and implications.

## Conclusion

This study explored the perspectives of South African rehabilitation healthcare stakeholders regarding the value of rehabilitation in the country. The perceived value of rehabilitation was found to be multifaceted, encompassing contextual factors, service delivery components, patient outcomes, economic considerations, and collaboration among diverse stakeholders. The unique challenges of the South African context, marked by diverse health, socio-economic, and geographic factors, contribute to its distinctive perspective on value. The study presents a prioritised framework of components crucial for delivering value-based rehabilitation services in South Africa, particularly considering the potential strengthening of rehabilitation within the healthcare system through the implementation of the NHI. Recommendations include exploring the perspectives of various additional stakeholders, such as patients, representatives from the disability and rehabilitation sectors, and the community, to achieve a more holistic understanding of the value of rehabilitation. Additionally, future research should focus on providing empirical evidence, including economic evaluations, to demonstrate the economic and societal value of rehabilitation.
